# Atlanta Medical College

**Published:** 1878-03

**Authors:** 


Atlanta Medical College.—The commencement exercises of this institution took place at night on the first of March, inst., in presence of a large audience.

The Dean's report showed the college in a prosperous condition, and more than usual emulation amongst members of the graduating class for prizes offered, and distinction for proficiency in a knowledge of the medical sciences. The graduating class was said to rank somewhat above the usual average.

The degree of M.D. was conferred by Col. W. L. Mitchell, of Athens, Georgia, one of the Board of Trustees, in his usual happy style, on twenty-four applicants, as follows:

George W. Blanton, George W. Burnett, Walter A. Camp, C. J. Church, Thomas M. Dean, B. L. Embry, John F. Fielder, A. B. Frix, J. E. Gillespie, E. T. Gauntt, I. J. Goodman, H. B. Harper, S. W. Johnson, W. R. K. Johnson, Lyman H. Jones, Lodrick M. Jones, Isaac Keeler, L. A. Lee, Thomas Northen, J. D. Orr, J. B. Reynolds, J. T. Selman, W. L. Wilkinson.

The valedictory on the part of the class was delivered by Dr. G. W. Blanton, of Dalton, in a very interesting manner, and responded to by Maj. R. N. Ely, Attorney-General of the State, to the satisfaction of all present.

Dr. J. H. Logan, Professor of Chemistry in the College, in a becoming manner awarded prizes as follows:

The prize offered by the faculty for the most thorough preparation in all the branches (a case of surgical instruments) was awarded to Dr. S. W. Johnson, of Cumming, Georgia; also, a medical case offered by the Professor of Materia Medica for the best thesis on the "Action of Remedies."

Honorable mention was made of Drs. J. B. Reynolds, Thomas Northen and Walter A. Camp as next in grade to the successful competitor.

A case of urethral instruments, offered by the Professor of Anatomy for the best thesis on "Stricture of the Urethra," was awarded to Dr. W. L. Wilkinson. A case of obstetrical instruments, offered by the Professor of Obstetrics for the best preparation in his branch, was awarded to Dr. W. A. Camp. A case of transfusion instruments, offered by the Professor of Surgery for the greatest proficiency in his branch, was awarded to Dr. J. B. Reynolds. An ophthalmoscope, offered by the Professor of Diseases of the Eye and Ear for the best thesis on "Diseases of the Eye," was awarded to Dr. W. A. Camp.

